# Effect of Thermoultrasound on the Antioxidant Compounds and Fatty Acid Profile of Blackberry (*Rubus fruticosus* spp.) Juice

**DOI:** 10.3390/molecules21121624

**Published:** 2016-11-29

**Authors:** José de Jesús Manríquez-Torres, José Antonio Sánchez-Franco, Esther Ramírez-Moreno, Nelly del Socorro Cruz-Cansino, José Alberto Ariza-Ortega, Jesús Martín Torres-Valencia

**Affiliations:** 1 Centro de Investigación Interdisciplinario. Área Académica de Nutrición, Instituto de Ciencias de la Salud, Universidad Autónoma del Estado de Hidalgo. Circuito Actopan-Tilcuautla s/n. Ex hacienda La Concepción. San Agustín Tlaxiaca, Hidalgo 42160, Mexico; baronruiz7@hotmail.com (J.A.S.-F.); rme1234@yahoo.com (E.R.-M.); ncruz@uaeh.edu.mx (N.d.S.C.-C.); jose190375@hotmail.com (J.A.A.-O.); 2 Área Académica de Química, Universidad Autónoma del Estado de Hidalgo, Km 4.5 Carretera Pachuca-Tulancingo, Mineral de la Reforma, Hidalgo 42184, Mexico; jmtv.np@gmail.com

**Keywords:** blackberry juice, thermoultrasound, antioxidant compounds, fatty acids

## Abstract

Blackberry (*Rubus fruticosus* spp.) fruit has high antioxidant activity due to its significant content of anthocyanins and antioxidant compounds. Among emerging technologies for food preservation, thermoultrasound is a technique that reduces microbial loads and releases compounds with antioxidant properties. The objective of this study was to determine the antioxidant content and fatty acid profile of blackberry juice subjected to thermoultrasound treatment in comparison to pasteurized juice. Blackberry juice and *n*-hexane extracts from a control (untreated juice), pasteurized, and thermoultrasonicated samples were evaluated for antioxidant activity, fatty acid profile, and antioxidant content. The juice treated with thermoultrasound exhibited significantly (*p* < 0.05) higher levels of total phenols (1011 mg GAE/L), anthocyanins (118 mg Cy-3-GlE/L); antioxidant activity by ABTS (44 mg VCEAC/L) and DPPH (2665 µmol TE/L) in comparison to the control and pasteurized samples. Oil extract from thermoultrasound juice also had the highest antioxidant activity (177.5 mg VCEAC/L and 1802.6 µmol TE/L). The fatty acid profile of the *n*-hexane extracts showed the presence of myristic, linolenic, stearic, oleic, and linoleic acids and was not affected by the treatments except for stearic acid, whose amount was particularly higher in the control. Our results demonstrated that thermoultrasound can be an alternative technology to pasteurization that maintains and releases antioxidant compounds and preserves the fatty acids of fruit juice.

## 1. Introduction

The blackberry fruit is a crop with high commercial value due to its sensory and chemical characteristics [1,2,3,4]. In Mexico blackberry is either consumed fresh (30%) or used as raw material (70%) for the production of jams, candy, ice cream, yogurt, wine, and juice beverages, among others [5,6]. The seeds of the blackberry fruit, which are typically removed during juice processing, have 6%–7% of protein, 11%–18% of oil with fatty acid contents of 53%–63% linoleic acid (C 18:2n-6), 15%–31% linolenic acid (C 18:3n-3), 3%–8% saturated fatty acids, and includes other phytochemicals [7]. Blackberry is also of great interest due to its high content of polyphenols. All of these compounds may benefit human health by reducing the risk of coronary heart disease and certain types of cancer [8].

Pasteurization is the conventional method used to decimate or kill bacteria in liquid foods, however the application of heat may reduce the content of nutrients in fruits and vegetables [9]. Emerging technologies are an alternative to heat treatment; ultrasound is one of them [10] with the potential to preserve several types of foods including liquids through the use of high-intensity sound waves, which generate physical disruptions and induce chemical reactions in the food matrix [11,12]. Ultrasound maintains quality parameters, such as color, flavor, and antioxidant properties and also facilitates the release of bioactive compounds [13]. However, ultrasound alone is not very effective, so it is combined with the application of moderately high temperatures (50–60 °C) in a treatment called thermoultrasound that prevents damage to the product by reducing the temperature and/or the time of treatment [14,15].

The objective of this study was to determine the impact of thermoultrasound, compared with the pasteurization, on the antioxidant properties and fatty acid profile of blackberry juice.

## 2. Results and Discussion

### 2.1. Antioxidant Activity of Juices

Different blackberry juices were developed: control (CTL), pasteurized (PAS), and thermoultrasonicated (TUS), obtaining a yield of 57% ± 1% of juice and 43% ± 1% of residue. Subsequently, juices were subjected to freeze-drying, obtaining a yield of extracts corresponding to each sample of about 1 g for every 10 mL of lyophilized juice. Table 1 shows the antioxidant properties of blackberry juice. Thermoultrasonicated samples exhibited a higher content of bioactive compounds and antioxidant activity compared to the control and pasteurized juice (*p* < 0.05). These results suggest that thermoultrasound maintained, and even increased, the release of total phenols, anthocyanins, and antioxidant activity of the juice.

#### 2.1.1. Effect of Thermoultrasound and Pasteurization on the Ascorbic Acid Content

The ascorbic acid content decreased in both treatments but was more pronounced in the thermoultrasonicated (24%) than in the pasteurized juice (9%) when compared to the control (Table 1). The observed slight decrease in pasteurized juice could be due to the oxidation of ascorbic acid caused by temperature (72 °C), whereas sonication may have induced oxidative processes in aerobic and anaerobic environments associated with the production and use of hydroxyl radicals [16,17].

#### 2.1.2. Effect of Thermoultrasound and Pasteurization on Total Phenolic and Anthocyanin Content

The thermoultrasonicated juice exhibited a greater release of total phenols (1011 mg GAE/L), which could be due to a synergistic effect between the temperature reached and the processing time exerted by the cavitation treatment (Table 1) [18]. Our results were similar to those reported for watermelon juice [18], in this study a lower temperature and longer processing time were used. The pasteurized juice had a reduction in phenolic compounds, which agrees with results reported for apple juice [19] that were attributed to the temperature of 80 °C for 15 min that degraded the polyphenolic compounds.

Regarding the anthocyanin content, the values were also significantly higher (*p* < 0.05) in the thermoultrasonicated juice (118.7 mg Cy-3-GlE/L) in contrast to the control and pasteurized samples (Table 1), which matches the extraction yields up to 20% of anthocyanins reported by other researchers for blackberry thermoultrasonicated juice [20].

#### 2.1.3. Effect of the Thermoultrasound and Pasteurization on the Antioxidant Activity of the Juices

A similar trend as that described above was observed for antioxidant activity by ABTS (2,2′-azino-bis(3-ethylbenzothiazoline-6-sulphonic acid)) (Table 1). The pasteurized juice presented a lower antioxidant activity (11.5 mg VCEAC/L) compared to the control (13.6 mg VCEAC/L) and the thermoultrasonicated juice (44.7 mg VCEAC/L). The antioxidant activity by ABTS in control juice was similar to that reported in previous studies [21]. Both treatments increased the antioxidant activity by DPPH (2,2-diphenyl-1-picrylhydrazyl), particularly the thermoultrasound treatment more than doubled the value obtained for control juice. Similar observations were reported by Zafra-Rojas et al. [13] who stated that the amplitude and time during an ultrasound treatment contributed to the release of antioxidants, and that the fruit juice antioxidant activity is comprised by the activities of compounds, such as phenolics, taurine, vitamins, betalains, anthocyanins, and ascorbic acid, among others, with known antioxidant properties [13].

### 2.2. Antioxidant Activity and Fatty Acid Profile of the n-Hexane Extracts from Blackberry Juice

Solvent extractions were performed with *n*-hexane using the lyophilized control, pasteurized and thermoultrasound juices and the yields obtained were 0.1%, 0.2%, and 0.1%, respectively.

#### 2.2.1. Effect of Thermoultrasound and Pasteurization on the Antioxidant Activity of the Extracts

The fractions obtained with the *n*-hexane extraction were analyzed by ABTS and DPPH to determine the treatment that best maintained the antioxidant properties of blackberry juice. The results are shown in Table 2. The higher values for both parameters ABTS and DPPH achieved by the thermoultrasound treatment can be attributed to the disruption of biological cell walls and, thus, the release of their antioxidant compounds [13].

#### 2.2.2. Fatty Acid Profile from Blackberry Juice

**Figure 1:**
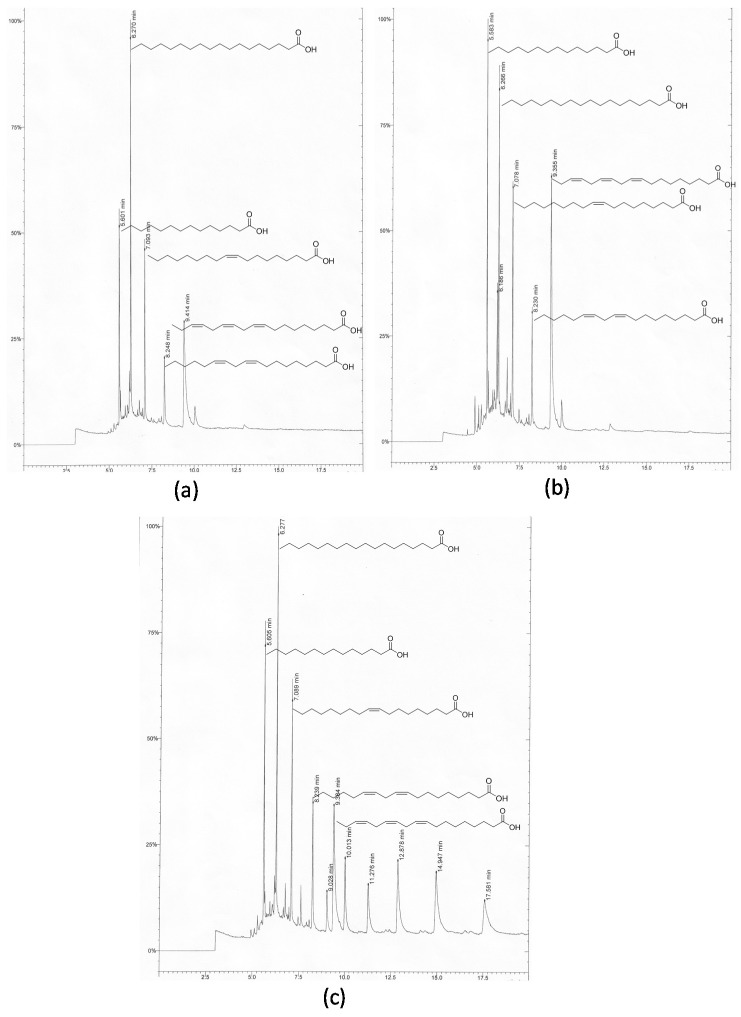
Chromatograms of the lipid profile of the hexane extractions performed in juices: (**a**) control juice; (**b**) pasteurized juice; and (**c**) thermoultrasonicated juice.

The fatty acid profiles of blackberry juice are described in Table 3 and Figure 1. The fatty acids identified and quantified in the control (untreated blackberry juice), pasteurized, and thermoultrasonicated juice were: saturated fatty acids myristic and stearic (C 14:0 and C 18:0, respectively), mono-unsaturated fatty acid oleic (C 18:1), and linoleic and linolenic poly-unsaturated fatty acids (C 18:2 and C 18:3). These compounds had been reported previously in fruits, such as *Rubus* spp., by Radocâj et al. [22]. Thermoultrasonication did not affect the concentration of fatty acids, except for stearic (C 18:0) and oleic (C 18:1) acids, which decreased in 42% and 23%, respectively, as compared to their content in the control juice; a similar trend was observed in pasteurized samples. Cavitation during ultrasound is likely the main cause of oxidation due to the formation and collapse of micro-bubbles resulting in areas of high temperature and pressure. In addition to thermal and shear force-induced oxidation, free radicals may also be generated by sonolysis through the increased release of metabolites [23,24,25], as suggested by the appearance of new signals shown in Figure 1c. The release of phytosterols that may be responsible for the highest antioxidant activity exhibited by the hexane extract from thermoultrasonicated juice (see Table 2), as has already been reported in previous studies [26].

## 3. Materials and Methods

### 3.1. Materials

Blackberries (*Rubus fruticosus* spp.) were obtained from a local market in Atotonilco, Hidalgo, México, during the winter of 2014. Folin–Ciocalteu 2 N reagent (Sigma-Aldrich, St. Louis, MO, USA), anhydrous sodium carbonate (Meyer, Tláhuac, DF, Mexico), gallic acid (Meyer), potassium chloride (Sigma-Aldrich), anhydrous sodium acetate (Meyer), hydrochloric acid (Reasol, Iztapalapa, DF, Mexico), 2,2′azino-bis(3-ethylbenzthiazoline-6-sulphonic acid) diammonium salt (ABTS) ≥ 98% (Sigma-Aldrich), potassium persulfate crystals (Meyer), Trolox 97% (Sigma-Aldrich), absolute ethanol (Meyer), 1,1-di-phenyl-2-picrylhydrazyl (DPPH) (Sigma-Aldrich). All chemicals and reagents used in the study were of analytical grade (AG).

### 3.2. Instruments

The following instruments were used in this study: an ultrasound (VCX-1500, Sonics and Materials, Inc., Newtown, CT, USA), industrial blender (38BL52 LBC10, Waring Commercial, Torrington, CT, USA), refrigerated centrifuge (Allegra 25™, Beckman Coulter, Palo Alto, CA, USA), lyophilizer (7753020, LABCONCO, Kansas City, MO, USA), analytical mill (IKA^®^ A11 basic, Wilmington, NC, USA), water bath (1210610, Cole-Parmer, Vernon Hills, IL, USA), overhead stirrer (HS-50A, Wisestir^®^ Wisd, laboratory instruments, Seongbuk-gu, Seoul, Korea), spectrophotometric microplate reader (Power Wave XS UV-Biotek, software Gen5 2.09, Winooski, VT, USA), and a Rotavapor^®^ R-300 (Buchi, Meierseggstrasse, Flawil, Switzerland).

### 3.3. Sample and Treatments

Only fruits without external injuries were selected and washed. Blackberry juice was obtained by stirring the fruit using an industrial blender (38BL52 LBC10, Waring Comercial, USA) and passed through a strainer to remove seeds and peels. Juice was then clarified by centrifugation at 10,000 rpm (Allegra 25™, Beckman Coulter, CA, USA) for 30 min at 4 °C. A sample of 400 mL was introduced and heated in a jacketed vessel filled with water at 45 ± 1°C; overheating of the sample was prevented by running cold water (Cole-Parmer) through the treatment chamber at 4 °C. Samples were treated by ultrasound (VCX-1500, Sonics and Materials, Inc., Newtown, CT, USA) at 1500 W, with a constant frequency of 20 kHz at 80% amplitude for 25 min with pulse duration of four seconds on and two seconds off using a 13 mm probe. Processing conditions (amplitude and time) were determined from a previous study in which the optimal treatment conditions for juice were established based on complete inactivation of microorganisms, and higher antioxidant capacity and content of phenolics and anthocyanins (1076.2 and 17,126.3 μmol TE/L, 3318.4 mg GAE/L, and 949.2 mg Cy-3-Gl/L, respectively) [20,27]. Untreated juice was used as control and other juice was pasteurized (70 °C, 30 min) and included to compare results. A solid phase extraction using a 3 cc-Oasis-HLBTM cartridge (Waters Corp., Milford, MA, USA) was carried out on the juices to eliminate interferences. The filtrate was used to determinate ascorbic acid, total phenol, anthocyanins and antioxidant activity by ABTS and DPPH. In the other series of experiments, the juices were frozen (−32 °C) and lyophilized (Labconco VWR26671-581 139 154, Kansas City, MO, USA), the extractions were performed subsequently with *n-*hexane in the control, pasteurized, and thermoultrasonicated juices. To obtain the extracts, 25 g of lyophilized juice were mixed with 250 mL of *n*-hexane, the mixture was filtered and concentrated using a rotavapor apparatus obtaining the following yields: 0.03 g, 0.04 g, and 0.02 g for control, pasteurized, and thermoultrasonicated juice, respectively. The obtained *n-*hexane extracts were analyzed for antioxidant activity by ABTS and DPPH, and fatty acid profile was determined by means of gas chromatography coupled to mass spectroscopy.

### 3.4. Determination of Ascorbic Acid Content

Ascorbic acid content was determined with 2,6-dichloroindophenol sodium salt hydrate (DCPI) and the sample was diluted in 0.4% oxalic acid. One-hundred microliters of juice was mixed with 100 µL of acetate buffer and 800 µL of DCPI. The absorbance of the mixture was measured at 520 nm in a microplate reader (Power Wave XS UV-Biotek, KC Junior software, Winooski, VT, USA). Ascorbic acid was used as a reference standard and the results were expressed as mg ascorbic acid per liter (mg AA/L) [28].

### 3.5. Determination of Total Phenolic Content

Total phenolic content of the juice was determined with the Folin-Ciocalteu reagent; 100 μL of sample was mixed with 500 μL of 1:10 diluted Folin-Ciocalteu reagent, then 400 μL (7.5%) of sodium carbonate was added and the mixture was incubated for 30 min at room temperature. The absorbance of the mixture was measured at 765 nm using a microplate reader (Power Wave XS UV-Biotek, KC Junior Software, USA). Gallic acid was used as a reference standard and the results were expressed as mg gallic acid equivalents per liter (mg GAE/L) [29].

### 3.6. Determination of Anthocyanins

The total monomeric anthocyanin content was measured according to the pH differential method [14]. Anthocyanin concentration was calculated based on the molecular weight (449.2) and extinction coefficient (26,900) for cyanidin-3-glucoside (Cy-3-Gl). The absorbance was measured at 510 and 700 nm in a microplate reader and the results were expressed as mg of Cy-3-Gl equivalent per liter (mg Cy-3-GlE/L).

### 3.7. Antiradical Capacity by ABTS^●+^

Antiradical capacity by ABTS was measured with the radical cation 2,2′-azino-bis (3-ethylbenzothiazoline-6-sulphonic acid) diammonium salt (ABTS^●+^), which was produced by reacting 7 mmol/L of ABTS^●+^ stock solution with 2.45 mmol/L potassium persulfate under dark conditions at room temperature for 16 h before use. The ABTS^●+^ solution was diluted with deionized water to an absorbance of 0.70 ± 0.10 read at 754 nm measured in the microplate reader (Power Wave XS UV-Biotek, KC Junior Software, USA). The results were expressed as mg Vitamin C equivalent antioxidant capacity per liter (mg VCEAC/L) of juice [30].

### 3.8. Antiradical Capacity by DPPH^●^

Antiradical activity was measured using 1,1-diphenyl-2-picrylhydrazyl (DPPH^●^) radical as described by Morales and Jiménez-Pérez [31]. The sample was diluted in deionized water (1:50). An ethanolic solution (7.4 mg/100 mL) of the stable DPPH^●^ radical was prepared. Then 100 μL of the sample was taken into vials and 500 μL of DPPH^●^ solution was added, and the mixture was left to stand at room temperature for 1 h. The solution was stirred and centrifuged at 3000 rpm during 10 min. Finally, absorbance was measured at 520 nm using a microplate reader (Power Wave XS UV-Biotek, KC Junior Software, USA) and the result was expressed as μmol trolox equivalent per liter (μmol TE/L).

### 3.9. Fatty Acids Profile by Gas Chromatography Coupled to Mass Spectroscopy

Fatty acids profiles of the *n*-hexane extracts obtained from the control, pasteurized, and thermoultrasonicated blackberry juice were identified by a gas chromatography detector. MS data were acquired in electron impact (EI) mode, and 70 electron volts, using a Hewlett Packard 5890 Series II spectrometer with a scan range of 20–601 *m*/*z*. Identification was confirmed by the retention time lock (RTL) of the fatty acids compared with a standard of 37 components (Food Industry FAMEs Mix, Restek, Bellefonte, PA, USA).The standard product used was a mix of methyl esters with chains C 4:0, C 6:0, C 8:0, C 10:0, C 11:0, C 12:0, C 13:0, C 14:0, C 14:1, C 15:0, C 15:1, C 16:0, C 16:1, C 17:0, C 17:1, C 18:0, C 18:1n-9, C 18:1n-9, C 18:2n-6, C 18:2n-6, C 18:3n-6, C 18:3n-3, C 20:0, C 20:1n-9, C 20:2, C 20:3n-6, C 20:3n-3, C 20:4n-6, C 20:5n-3, C 21:0, C 22:0, C 22:1n-9, C 22:2, C 22:6n-3, C 23:0, C 24:0, and C 24:1n-9 to compare the fragmentation patterns. All determinations were carried out in triplicate.

### 3.10. Statistical Analysis

All values were obtained by triplicate and expressed as mean ± standard deviation (SD). Data were analyzed by one-way analysis of variance (ANOVA), and differences among means were determined using the Tukey test with a level of significance of *p* < 0.05. The statistical package SPSS^®^ (Chicago, IL, USA) System for WINTM version 15.0 was used.

## 4. Conclusions

Thermoultrasound is a technique enabling the extraction of active compounds while having a minor impact on other bioactive molecules as compared to other conventional techniques, such as pasteurization. Thermoultrasound yielded a higher antioxidant activity than control samples and our results demonstrated that it is a valuable technology for juice processing because, in addition to the increase the antioxidant activity, it also retained the nutritional properties of the blackberry juice.

## Figures and Tables

**Table 1:** Antioxidants content and antioxidant activity in blackberry juices.

Determination	CTL	PAS	TUS
Ascorbic acid (mg AA/L)	27.7 ± 0.8 ^c^	25.2 ± 0.8 ^b^	21.3 ± 0.6 ^a^
Total phenols (mg GAE/L)	726.2 ± 4.8 ^a^	789.6 ± 3.9 ^b^	1011.6 ± 3.9 ^c^
Anthocyanins (mg Cy-3-GlE/L)	106.3 ± 1.3 ^b^	94.4 ± 4.2 ^a^	118.7 ± 1.4 ^c^
ABTS (mg VCEAC/L)	13.6 ± 0.2 ^b^	11.5 ± 0.4 ^a^	44.7 ± 1.2 ^c^
DPPH (µmol TE/L)	1146.5 ± 1.9 ^a^	1319.8 ± 1.0 ^b^	2655.9 ± 14.0 ^c^

^a–c^ Different letters in the same line indicate significant difference (*p* < 0.05) between the juices. AA: ascorbic acid; GAE: gallic acid equivalents; TE: trolox equivalent; VCEAC: vitamin C equivalent antioxidant capacity; Cy-3-Gl: cyanidin-3-glucoside equivalent; CTL: control; PAS: pasteurized; TUS: thermoultrasound.

**Table 2:** Antioxidant activity of the *n-*hexane extracts from blackberry juices.

Determination	CTL	PAS	TUS
ABTS (mg VCEAC/L)	63.7 ± 1.1 ^b^	12.1 ± 1.0 ^a^	177.5 ± 0.7 ^c^
DPPH (µmol TE/L)	445.2 ± 1.6 ^b^	99.5 ± 3.0 ^a^	1802.6 ± 1.1 ^c^

^a–c^ Different letters in the same line indicate significant difference (*p* < 0.05) between the juices. TE: trolox equivalent; VCEAC: vitamin C equivalent antioxidant capacity; CTL: control; PAS: pasteurized; TUS: thermoultrasound.

**Table 3:** Fatty acid composition of the oil extracted with hexane from experimental blackberry juices.

Fatty Acid (% *w*/*w*)	Blackberry Juice Oil		
	Control	Pasteurized	Thermoultrasonic
C 14:0	21.8 ± 0.1 ^b^	23.8 ± 0.6 ^c^	18.0 ± 0.7 ^a^
C 18:0	40.5 ± 0.3 ^c^	21.1 ± 0.4 ^a^	23.2 ± 0.4 ^b^
C 18:1	19.1 ± 0.2 ^c^	14.6 ± 0.4 ^a^	14.7 ± 0.3 ^b^
C 18:2n-6	7.7 ± 0.4 ^b^	7.3 ± 0.1 ^a^	7.8 ± 0.7 ^c^
C 18:3n-3	11.0 ± 0.5 ^b^	14.9 ± 0.6 ^c^	7.6 ± 0.6 ^a^

^a–c^ Different letters in the same line indicate significant difference (*p* < 0.05) between the juices in the same determination. Values are means SD; *n* = 3.
